# Impact of ambient air pollution on physical activity and sedentary behavior in children

**DOI:** 10.1186/s12889-023-15269-8

**Published:** 2023-02-17

**Authors:** Hongjun Yu, Heran Zhang

**Affiliations:** 1 Department of Physical Education, Tsinghua University, Tsinghua Yuan Str, Beijing, 100084 China; 2 Winter Sports Administrative Center of General Administration of Sport of China, Beijing, China

**Keywords:** AQI, PM_2.5_, PM_10_, Physical activity, Sedentary behavior, Children

## Abstract

**Background:**

Exposure to air pollution has become a serious environmental issue affecting children’s health and health-related behavior in China. Previous studies have focused on the associations between air pollution and physical activity among adults; however, few have examined the relationship between air pollution and health-related behavior among children, which are particularly susceptible population subgroups. The present study aims to examine the impact of air pollution on daily physical activity (PA) and sedentary behavior (SB) among children in China.

**Methods:**

PA and SB data were collected by actiGraph accelerometers for eight consecutive days. PA and SB data from 206 children were matched to daily air pollution obtained from the Ministry of Environmental Protection of the People’s Republic of China, including the average daily air quality index (AQI), PM_2.5_ (µg/m³), and PM_10_ (µg/m³). Associations were estimated using linear individual fixed-effect regressions.

**Results:**

A 10-unit increase in daily AQI was associated with a reduction in daily PA by 5.94 (95% confidence interval [CI] = -8.79, -3.08) minutes of moderate to vigorous physical activity (MVPA) and 229.82 (95% CI = -345.35, -114.28) walking steps and an increase in daily SB by 15.77 (95% CI = 9.01, 22.53) minutes. A 10 µg/m³ increase in air pollution concentration in daily PM_2.5_ was associated with a reduction in daily PA by 7.51 (95% CI = -11.04, -3.97) minutes of MVPA, 295.69 (95% CI = -438.46, -152.92) walking steps and an increase in daily SB by 21.12 (95% CI = 12.77, 29.47) minutes. A 10 µg/m³ increase in air pollution concentration in daily PM_10_ was associated with a reduction in daily PA by 13.18 (95% confidence interval [CI] = -15.98, -10.37) minutes of MVPA, 518.34 (95% CI = -631.77, -404.91) walking steps and an increase in daily SB by 19.87 (95% CI = 13.10, 26.64) minutes.

**Conclusion:**

Air pollution may discourage physical activity and increase sedentary behavior among children. Policy interventions are needed to reduce air pollution and develop strategies to decrease risks to children’s health.

## Introduction

Air pollution has been identified as a major environmental health risk factor for non-communicable diseases such as ischaemic heart disease, stroke, chronic obstructive pulmonary disease and asthma [1, 2]. World Health Organization (WHO) estimated that 99% of the global population breathes air whose quality exceeds WHO guideline limits, resulting in 4.2 million premature deaths every year due to exposure to outdoor air pollution [3]. Children, older adults, and patients with lung disease are most susceptible to the effects of air pollution [4]. Thus, on days with high air pollution levels, adults, especially children and older adults, who are the most vulnerable population, are expected or encouraged to avoid outdoor activities, travel by public transport, or stay at home to minimize the risk of exposure [4, 5].

Physical inactivity has caused more than 5 million deaths globally each year according to investigations by WHO [6]. Furthermore, the WHO also reports that global estimates approximately 25% of adults and 81% of adolescents do not achieve the recommended guidelines [6]. For instance, the WHO recommends that children under 5 years old should engage in various physical activities at any intensity for at least 180 min [7]. Previous studies also reported the health benefits of regular physical activities for children under 5 years old, including improving adiposity, cardiometabolic health, bone and skeletal health, and cognitive and motor skills development [8, 9].

In recent years, China has been prioritizing several measures to minimize its population’s exposure to air pollution [10, 11]. However, schoolyards are common places to do physical activities for children and adolescents [12]. Our previous studies have shown that air pollution in China may further discourage young adults and older adults from engaging in regular physical activities and exercises [13, 14]. Several studies have examined the impact of ambient air pollution on physical activity and sedentary behavior among adults [15] and older adults [16]. A recent mapping review located 14 studies which examined the associations between air pollution on physical activity [4]. With respect to the aforementioned work, we found that four major problems remained unclear. First, few studies were performed among children who were potentially susceptible to air pollution. Among these studies, 11 were performed among adults [14, 15, 17–25], and only 3 were performed among older adults [13, 16, 25]. To our knowledge, no study has reported the impact of ambient air pollution on physical activity and sedentary behavior among children. Second, most previous studies adopted a cross-sectional study design [15, 18–22, 24, 25]. There are only five longitudinal studies that have reported air pollution’s impact on college students [14, 17, 26] and older adults [13, 16]. Third, most previous studies performed self-reported physical activity [13–15, 17–22, 24, 25]. There are only three studies which used objective methods, one used iPhone apps [21], and two used accelerometers [13, 26, 27]. Fourth, to date, previous studies estimated the impact of air pollution on physical activity and sedentary behaviors week by week [13, 14, 17], month by month [18], or year by year [15, 22]. No study investigates the relationship at day by day level.

In the current study, we examined the impact of air pollution on physical activity and sedentary behavior among children in China. Objectively measured data were collected from 206 children by using digital accelerometers during this study. In the prospective cohort study, we measured daily changes in air quality for eight consecutive days and examined daily variations in children’s physical activities and sedentary behaviors. We hypothesized that children’s physical activities would be reduced due to air pollution, while children’s sedentary behaviors would be increased accordingly.

## Methods

### Participants and sampling procedure

This study recruited 212 children in Beijing Fortune Fountain Kindergarten (Yayuan and Ziteng Garden Dachang Hui Autonomous County, Langfang City, Hebei Province, China). The study was conducted from December 2019 to December 2020. And the children were recruited during several periods (Dec 24–31, 2019; Jan 7–14, 2020; Nov 17–25, 2020; Dec 3–11, 2020; Dec 15–23, 2020).

The COVID-19 confinement led to a reduction in the physical activity of people in China [28]. Since the outbreak of the new crown epidemic in China, as of on January 26, 2020, 30 provinces in China imposed lockdown policy and on April 8, 2020, China released lockdown policy. From August 2021 to the present, China initiated zero-COVID policy. However, the children were recruited during the period of end COVID-19 lockdown policy in China. Therefore, the Chinese government lockdown policy did not impact children’s behavior in our study.

**Fig. 1:**
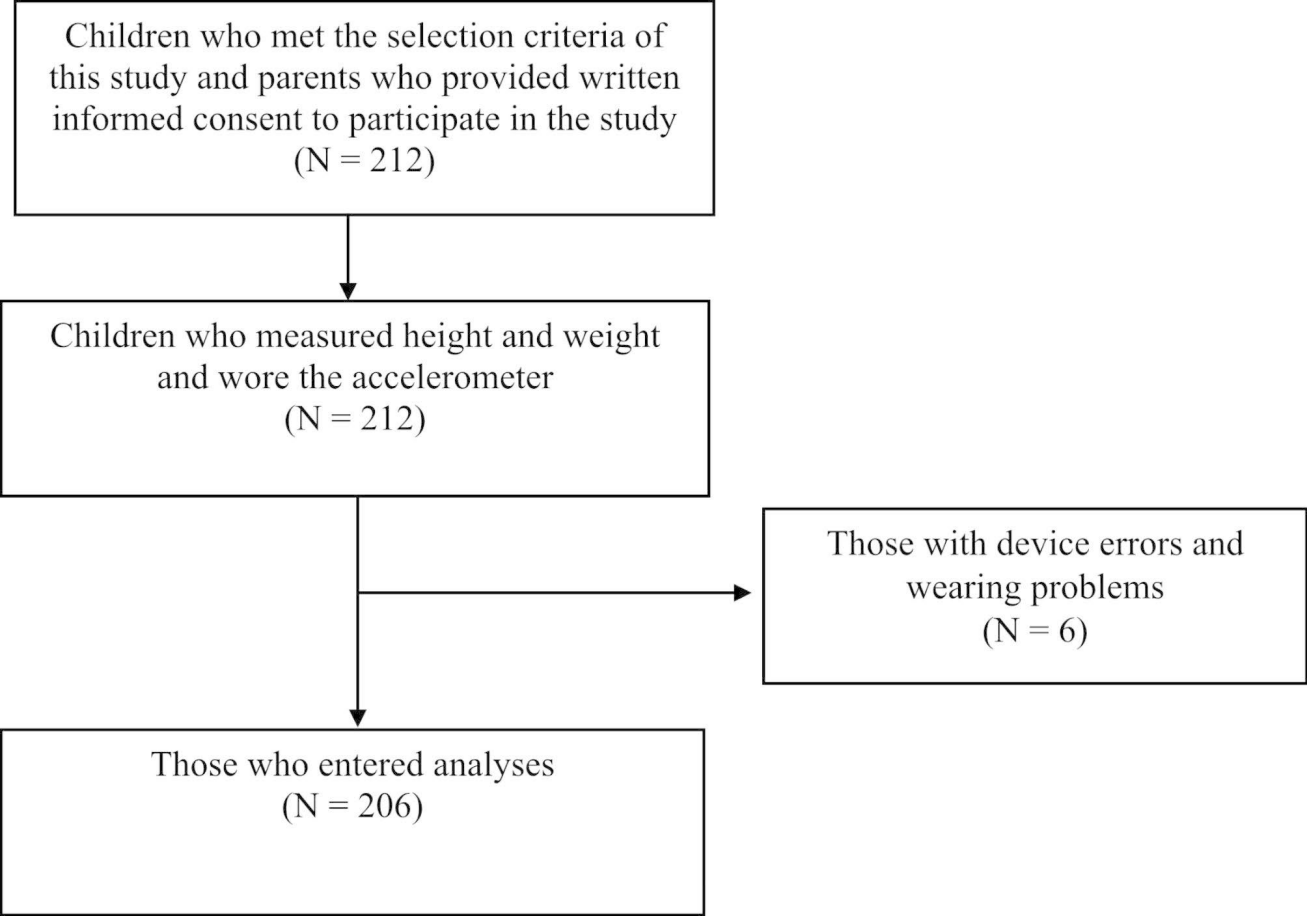
Study Sample Flowchart

Principals, teachers and parents provided written informed consent to participate in the study. Upon acceptance, the subjects were asked to visit a classroom to get a wGT3X-BT (ActiGraph company) device fitted. The height of the child was measured by using a height meter, and the accuracy was controlled by 0.1 cm. The test children kept their bodies upright on the measuring plate, with their arms at sides, heads up straight, heels closed, and toes separated. The height data were recorded in cm, with 1 decimal place reserved. The weight was measured using a lever scale (RGT-60-RT). The child stood in the middle of the scale, and the value was read after the child was stable. The weight data were recorded in kilograms, with 1 decimal place reserved. Before the initiation of this study, we sent recruitment information to the kindergarten classes. Parents who were interested were asked to complete a survey on children’s medical history and demographical information in the form of one paper-pencil-based health questionnaire on medical history, age, gender, ethnicity, physical activity habits, and mental and physical health conditions. The inclusion criteria of this study were that: (1) ages between 4 and 6; (2) free from diseases or any medical conditions; (3) obtained written informed consent to participate in the study from parents. Among the 212 children who had completed the study, 6 subjects were excluded due to device errors (4 subjects) and device wearing problems (2 subjects), thus presenting the data of 206 participants. Figure 1 presents the analytic sample selection flowchart for this study. In the present longitudinal study, we analyzed data collected for eight consecutive days to analyze the impact of daily air quality changes on children’s day-to-day physical activity during the research period. This project was approved by the Tsinghua University Institutional Review Board (IRB #2017DX02_11).

### Measures

#### Environmental measures

We obtained air quality data from Cnemc website of the air quality (http://www.cnemc.cn/), which is a site closest to the preschools. The data collection site is Da Chang air quality monitoring station. All participants’ residential addresses and kindergarten addresses were in Da Chang county town, which is approximately 7 KM from the county town center. For the dates, each student wore an ActiGraph accelerometers. Air pollution data and other environmental data were recorded, including the air quality index (AQI), PM_2.5_, PM_10_ average daytime temperature (C), and percentage of rainy days. China Meteorological Administration calculates the AQI for five measures of air quality: Ozone, particulate matter (< 2.5 µg and < 10 µg), carbon monoxide, sulfur dioxide, and nitrogen dioxide. The AQI value is calculated hourly, SO_2_, NO_2_, CO concentrations are measured as average per 24 h, O_3_ concentration is measured as average per hour and the moving average per 8 h, PM_2.5_ and PM_10_ concentrations are measured as average per hour and per 24 h. The AQI is an index for reporting daily air quality, including the condition of the surrounding air and alerts for associated health concerns. The details of AQI can be accessed elsewhere [26]. The mean values of 24-hour data of AQI, PM_2.5_, and PM_10_ were provided by Municipal Ecological Environment Bureau. The air quality measurements are concurrent with accelerometer measurements.

#### PA and SB measurement

PA and SB were measured using ActiGraph wGT3xBT triaxial accelerometer (www.actigraphcorp.com). ActiGraph wGT3x-BT was validated using the gold standard by Doubly labeled water (DLW) method in children [29]. The monitors were set to collect data at 50 Hz. The children were instructed to wear wGT3X-BT accelerometer on the left wrist for at least 10 h a day for eight consecutive days. The children were instructed to remove the device only when they were showering, bathing, swimming, or participating in other water-related activities. The recording epoch was set to record by 10 s. Daily physical activity and sedentary behavior were calculated using the minutes’ data. For all subjects, absolute time in moderate-to-vigorous (MVPA), light physical activity, walking steps, kcals in energy expenditure, and absolute time spent in sedentary behavior were estimated using the device. Nonwearing time was defined as 60 consecutive minutes of zero activity intensity count at 1 METs and 0 kcals in one hour according to Troiano et al. [30]. The minimum wear time for data to be included in the analysis was 10 h each day for at least four days, including one weekend day [31]. ActiLife software (version 6.13.4 ActiGraph corporation) was used to process the raw data to derive a filtered sum of vector magnitudes (VM) in 10-second epochs. And the time spent in all activities in different intensity-specific PA levels and SB were classified into SB, light PA, MVPA for each child. The cut points were defined for SB as VM ≤ 305, for light PA as VM 306–817, and for MVPA as VM ≥ 818 according to Chandler et al. [32].

### Statistical methods

We examined data for outliers and computed descriptive statistics including means, SD, and percentage for each variable stratified by gender. T-tests were used to compare continuous variables. Our main aim was to analyze air pollution variation and PA among Children. The key independent variables were AQI [33], PM_2.5,_ and PM_10_. We observed the changes by 10 units in AQI, PM_2.5_ and PM_10_ in fixed effect model analysis. Analyses were adjusted for covariates average daytime temperature and rain, age, self-rated physical health (on a scale of 1 to 10, worst to best by parents), self-rated mental health (on a scale of 1 to 10, poor to excellent by parents), temporal order and BMI. All participants were grouped by sex for subsequent statistical analysis.

The fixed-effects model or the random-effects model was performed in the panel analysis. Hausman test was performed to compare the differences in coefficient estimates under the two specifications. By performing individual fixed-effect regression, the fix-effects specification was estimated over and to be more suitable for the same sample used for the random-effects estimation. Compared to random effects or multivariate analysis of variance (MANOVA), individual fixed-effect regression model that treats individuals as their own control is preferred [34]. This study only focuses on within-person variations, and all between-person and time-invariant variations are conditioned out of the model [34]. Individual fixed-effect model removing potential omitted variable bias due to differences in time-invariant individual characteristics such as genes, ethnicity, habits, and personal preferences. We calculated all fixed-effect models in the Stata 17.0.

## Results

### Descriptive statistics

Descriptive statistics for 206 recruited children stratified by gender are presented (Table 1). 48% of participants were boys. Participants were compliant wearing GT3X accelerometer 1538 days over 8 continuous days. Compliance with wearing the accelerometers was 7.75 days (SD = 1.04) for boys and 7.69 (SD = 1.26) for girls. The mean compliant wearing hours in one day of the participants was 21.58 (SD = 4.79). The mean age of the participants was 4.55 years (SD = 1.03). The mean children’s BMI was 15.89 kg/m^2^ (SD = 3.20).

**Table 1:** The characteristics of subjects

Characteristics	Male	Female	Total	p
Gender, n (%)	98 (47.57)	108 (52.42)	206	
Compliance, total days	742 (48.24)	796 (51.76)	1,538	
Compliance days, mean (SD)	7.75 (1.04)	7.69 (1.26)	7.72 (1.16)	0.139
Age (yr), mean (SD)	4.44 (0.99)	4.66 (1.06)	4.55 (1.03)	0.128
Height (cm), mean (SD)	109.05 (7.60)	108.39 (7.22)	108.70 (7.40)	0.523
Weight (kg), mean (SD)	19.11 (3.29)	18.64 (3.12)	18.86 (3.20)	0.299
Body mass index, mean (SD)				
BMI (kg/m^2^)	15.98 (3.29)	15.80 (3.12)	15.89 (3.20)	0.407

### The air pollution variations

The variations in air quality measures were presented during the study period (Table 2). The mean AQI value at days was 42.85 (SD = 28.10). There were 1,076 (69.69%), 368 (23.93%), 79 (5.14%), 15 (0.97%) one day (24 h) AQI at “Good” AQI (0–50), “Moderate” AQI (51–100), “Unhealthy for sensitive groups” AQI (101–150), “Unhealthy” AQI (151–200), respectively. The average 24-hour PM_2.5_ exposure level was 20.00 µg/m^3^ (SD = 23.77). A majority proportion (57.24%) of average PM_2.5_ exposure levels exceeded the WHO recommended short-term (24-hour) air quality guidelines (AQG) level and interim targets for the PM_2.5_ standard of 15 µg/m^3^. The average 24-hour PM_10_ exposure level was 40.02 µg/m^3^ (SD = 25.40). A majority proportion (71.07%) of average PM_10_ exposure levels were under the WHO-recommended short-term (24-hour) AQG level for the PM_10_ standard of no more than 45 µg/m^3^. The mean temperature was − 1.77 °C (SD = 2.84). A majority proportion (95.45%) of days were sunny days.

**Table 2:** Air quality index, PM_2.5 and PM10_ categories during the study period by day

	Level	Alarm	Levels of health concern	Value range	N	%
AQI					1,538	100
	1	Green	Good	0–50	1,076	69.96
	2	Yellow	Moderate	50.1–100	368	23.93
	3	Orange	Unhealthy for sensitive groups	100.1–150	79	5.14
	4	Red	Unhealth	150.1–200	15	0.97
PM_2.5_ (µg/m^3^)					1,538	100
	1	Green	Good	1–15	673	43.76
	2	Green	Good	15.1–25	288	18.72
	3	Yellow	Moderate	25.1–37.5	201	13.07
	4	Orange	Unhealthy for sensitive groups	37.6–50	237	15.41
	5	Red	Unhealth	50.1–75	61	3.97
	6	Purple	Very unhealthy	75.1–150	78	5.07
PM_10_ (µg/m^3^)					1,538	100
	1	Green	Good	11–45	1,093	71.07
	2	Green	Good	45–50	109	7.09
	3	Yellow	Moderate	50.1–75	233	15.15
	4	Orange	Unhealthy for sensitive groups	75.1–100	19	1.23
	5	Red	Unhealth	100.1–150	84	5.46
Temperature (°C)					1,538	100
	1			-9.5–5	174	11.31
	2			-4.9-0	1,193	77.57
	3			0.1-5	121	7.87
	4			6–11	50	3.25
					1,538	100
Rain (%)	Yes				70	4.55
	No				1,468	95.45

### The PA and SB variations

The main descriptive PA and SB results for boys and girls were presented (Table 3). Overall, boys and girls had no statistically significant differences in MVPA. The mean minutes of the children’s one-day MVPA were 246.81 (SD = 121.70). However, boys had statistically significantly higher activity levels than girls in very vigorous PA (p < .001). As illustrated, the mean children’s kcals of energy expenditure in one day were 185.03 (SD = 152.48). Whereas boys had statistically significantly higher energy expenditure than girls in kcals (p < .01). No other statistically significant differences were measured when we compared light PA, steps, and SB in one day by gender. The mean minutes of the children’s one-day light PA were 232.89 (SD = 108.10). The mean steps of the children’s one-day walking were 10,138.28 (SD = 4,877.72). Whereas the mean hours of children’s one-day SB were 13.59 (SD = 4.58).

**Table 3:** Average physical activity and sedentary behavior

Dependent variables	Male	Female	Total	p
Compliance (daily hours), mean (SD)	15.67 (8.96)	15.66 (8.89)	15.67 (8.93)	0.487
MVPA (daily minutes), mean (SD)	245.08 (118.99)	248.42 (124.22)	246.81 (121.70)	0.705
Moderate PA (daily minutes), mean (SD)	188.52 (89.33)	194.05 (94.60)	191.38 (92.11)	0.880
Vigorous PA (daily minutes), mean (SD)	42.10 (24.34)	41.88 (24.59)	41.99 (24.46)	0.432
Very vigorous PA (daily minutes), mean (SD)	14.46 (12.51)	12.49 (11.21)	13.44 (11.89)	< 0.001
Light PA (daily minutes), mean (SD)	229.99 (105.72)	235.59 (110.28)	232.89 (108.10)	0.845
Sedentary PA(daily minutes), mean (SD)	825.41 (271.00)	806.02 (277.70)	815.37 (274.57)	0.083
Steps, mean (SD)	10,118.83 (4,818.58)	10,156.41 (4,935.16)	10,138.28 (4,877.72)	0.560
Kcals, mean (SD)	196.77 (160.76)	174.09 (143.57)	185.03 (152.48)	0.002

### The relationship between air pollution and PA, SB

#### Impact of AQI on PA and SB

The measured associations of air quality index (AQI) on individual-level outcomes of daily PA of different intensities (i.e., light PA, MPA, MVPA) and SB using linear individual fixed-effect regressions were presented (Table 4). AQI was found to be significantly negatively associated with one-day PA among children. A 10-unit increase in AQI was linked with a significant reduction in minutes of one-day MVPA, light PA, walking steps, in kcals of one-day energy expenditure by -5.94 (95% confidence interval [CI] = -8.79, -3.08), -4.73 (95% confidence interval [CI] = -7.25, -2.22), -229.82 (95% confidence interval [CI] = -345.35, -114.28) and - 3.93 (95% confidence interval [CI] = -7.03, -0.82) respectively (p < .001). AQI was found to be significantly positively associated with one-day SB among children. A 10-unit increase in AQI was linked with a significant increase in minutes of one-day SB by 15.77 (95% confidence interval [CI] = 9.01, 22.53). Similar relationships between air pollution and PA, and SB were found for girls and boys. The estimated decline results in response to an elevated AQI for girls engaged in MVPA and walking steps might have higher than for boys.

**Table 4:** Estimated associations of AQI, PM_2.5,_ and PM_10_ on individual-level physical activity outcomes by sex

	Dependent variable	Total		Male only		Female only	
		Coefficient (95% CI)	# Observations (# participants)	Coefficient (95% CI)	# Observations (# participants)	Coefficient (95% CI)	# Observations (# participants)
**AQI**							
	Total minutes of daily MVPA	-5.94*** (-8.79, -3.08)	1,538 (206)	-4.66* (-8.65, -0.67)	742 (98)	-7.15** (-11.25, -3.04)	796 (108)
	Total minutes of daily moderate physical activity	-4.42*** (-6.58, -2.25)	1,538 (206)	-3.23* (-6.22, -0.23)	742 (98)	-5.55** (-8.68, -2.42)	796 (108)
	Total minutes of daily vigorous physical activity	-1.19*** (-1.74, -0.63)	1,538 (206)	-0.97* (-1.76, -0.18)	742 (98)	-1.40** (-2.18, -0.61)	796 (108)
	Total minutes of daily very vigorous physical activity	-0.33** (-0.58, -0.08)	1,538 (206)	-0.47* (-0.85, -0.09)	742 (98)	-0.20 (-0.53, 0.13)	796 (108)
	Total minutes of daily light physical activity	-4.73*** (-7.25, -2.22)	1,538 (206)	-3.60* (-7.18, -0.01)	742 (98)	-5.82** (-9.36, -2.27)	796 (108)
	Total minutes of daily sedentary physical activity	15.77*** (9.01, 22.53)	1,538 (206)	15.06** (5.52, 24.61)	742 (98)	16.43** (6.82, 26.04)	796 (108)
	Total steps of daily walk	-229.82*** (-345.35, -114.28)	1,538 (206)	-182.75* (-346.15, -19.35)	742 (98)	-274.17** (-437.94, -110.39)	796 (108)
	Total daily kcals	-3.93* (-7.03, -0.82)	1,538 (206)	-5.24* (-9.98, -0.51)	742 (98)	-2.65 (-6.72, 1.42)	796 (108)
**PM**_**2.5**_							
	Total minutes of daily MVPA	-7.51*** (-11.04, -3.97)	1,538 (206)	-5.77* (-10.69, -0.86)	742 (98)	-9.19*** (-14.27, -4.10)	796 (108)
	Total minutes of daily moderate physical activity	-5.35*** (-8.03, -2.67)	1,538 (206)	-3.82* (-7.51, -0.14)	742 (98)	-6.82** (-10.70, -2.94)	796 (108)
	Total minutes of daily vigorous physical activity	-1.55*** (-2.24, -0.87)	1,538 (206)	-1.20* (-2.17, -0.23)	742 (98)	-1.89*** (-2.87, -0.92)	796 (108)
	Total minutes of daily very vigorous physical activity	-0.61*** (-0.92, -0.30)	1,538 (206)	-0.75** (-1.22, -0.27)	742 (98)	-0.47*** (-0.88, -0.07)	796 (108)
	Total minutes of daily light physical activity	-5.84*** (-8.95, -2.72)	1,538 (206)	-4.64* (-9.06, -0.22)	742 (98)	-7.00** (-11.41, -2.60)	796 (108)
	Total minutes of daily sedentary physical activity	21.12*** (12.77, 29.47)	1,538 (206)	19.17** (7.42, 30.92)	742 (98)	23.03*** (11.13, 34.92)	796 (108)
	Total steps of daily walk	-295.69*** (-438.46, -152.92)	1,538 (206)	-229.75*** (-432.03, -28.48)	742 (98)	-359.33** (-562.33, -156.33)	796 (108)
	Total daily kcals	-7.06*** (-10.88, -3.23)	1,538 (206)	-8.48** (-14.29, -2.66)	742 (98)	-5.68* (-10.72, -0.65)	796 (108)
**PM**_**10**_							
	Total minutes of daily MVPA	-13.18*** (-15.98, -10.37)	1,538 (206)	-11.04*** (-15.01, -7.07)	742 (98)	-15.15*** (-19.11, -11.18)	796 (108)
	Total minutes of daily moderate physical activity	-10.12*** (-12.24, -8.00)	1,538 (206)	-8.13*** (-11.10, -5.15)	742 (98)	-11.96*** (-14.98, -8.94)	796 (108)
	Total minutes of daily vigorous physical activity	-2.44*** (-2.99, -1.90)	1,538 (206)	-2.17*** (-2.96, -1.38)	742 (98)	-2.69*** (-3.45, -1.93)	796 (108)
	Total minutes of daily very vigorous physical activity	-0.61*** (-0.86, -0.36)	1,538 (206)	-0.74*** (-1.13,-0.36)	742 (98)	-0.49** (-0.82, -0.16)	796 (108)
	Total minutes of daily light physical activity	-10.96*** (-13.43, -8.48)	1,538 (206)	-8.61*** (-12.19, -5.03)	742 (98)	-13.13*** (-16.55, 9.70)	796 (108)
	Total minutes of daily sedentary physical activity	19.87*** (13.10, 26.64)	1,538 (206)	18.58*** (8.95, 28.21)	742 (98)	21.05*** (11.51, 30.59)	796 (108)
	Total steps of daily walk	-518.34*** (-631.77, -404.91)	1,538 (206)	-442.98*** (-605.58, -280.38)	742 (98)	-587.58*** (-746.29, -428.87)	796 (108)
	Total daily kcals	-8.33*** (-11.42, -5.23)	1,538 (206)	-9.38*** (-14.14, -4.62)	742 (98)	-7.33*** (-11.36, -3.30)	796 (108)

Notes: Separate individual fixed-effect regressions were performed to estimate the associations of air pollution concentrations on samples stratified by sex. Models adjust for all time-variant individual characteristics listed in Table 1 (i.e., age, BMI, self-rated physical health, and self-rated mental health), temporal order for participants and environmental variables listed in Table 3 (average daily temperature and percentage of rainy day in last week). *_p_ < *.05;* **_p_ < *.01;* ***_p_ < *.001*

#### Impact of PM_2.5_ on PA and SB

The estimated relationship between air pollution concentration in PM_2.5_ and individual-level outcomes of daily PA of different intensities (i.e., light PA, MVPA) and SB. PM_2.5_ was found to be significantly negatively associated with one-day PA among participants (Table 4). A 10 µg/m³ increase in PM_2.5_ was associated with a significant reduction in minutes of one-day MVPA, light PA, and walking steps, in kcals of one-day energy expenditure by -7.51 (95% confidence interval [CI] = -11.04, -3.97), -5.84 (95% confidence interval [CI] = -8.95, -2.72), -295.69 (95% confidence interval [CI] = -438.46, -152.92) and - 7.06 (95% confidence interval [CI] = -10.88, -3.23), respectively (p < .001). PM_2.5_ was found to be significantly positively associated with one-day SB among children. A 10 µg/m³ increase in PM_2.5_ was linked with a significant increase in minutes of one-day SB by 21.12 (95% confidence interval [CI] = 12.77, 29.47).

Similar relationships between air pollution and PA, and SB were found for girls and boys. The estimated decline results in response to an elevated PM_2.5_ for girls engaged in MVPA and walking steps are higher than for boys.

#### Impact of PM_10_ on PA and SB

The estimated associations of air pollution concentration in PM_10_ on individual-level outcomes of daily PA and SB were indicated (Table 4). PM_10_ was found to be significantly negatively associated with one-day PA (i.e., light PA, MVPA) among participants. A 10 µg/m³ increase in PM_10_ was associated with a significant reduction in minutes of one-day MVPA, light PA, one-day walking steps, in kcals of one-hour energy expenditure by -13.18 (95% confidence interval [CI] = -15.98, -10.37), -10.96 (95% confidence interval [CI] = -13.43, -8.48), -518.34 (95% confidence interval [CI] = -631.77, -404.91) and − 8.33 (95% confidence interval [CI] = -11.42, -5.23), respectively (p < .001). A 10 µg/m³ increase in PM_2.5_ was linked with a significant increase in minutes of one-day SB by 19.87 (95% confidence interval [CI] = 13.10, 26.64). Likely, the estimated associations of PA and SB were similar between boys and girls. The estimated decline in response to an elevated PM_2.5_ for girls in minutes of one-day MVPA and in one-day walking steps are had higher than for boys.

## Discussion

This study examined the impact of air pollution levels on physical activity and sedentary behavior among children in China using objectively-measured ways. The air pollution level was found to be negatively associated with daily physical activity but positively associated with daily sedentary behavior. To our best knowledge, this is the first study to estimate air pollution on physical activity and sedentary behavior for children. In addition, this is the first study to perform an accelerometer to estimate the impact of daily air pollution on daily physical activity and sedentary behavior. This study reveals that air pollution leads to a decrease in physical activity behavior and an increase in sedentary behavior in children.

In our study, air pollution was negatively linked with physical activity for children. Our results show an increased level in daily AQI, ambient PM_2.5_, and ambient PM_10_, which were associated with a reduction in light physical activity, MVPA, and walking steps counts per day. This finding is consistent with our previous research [14, 26] estimating the association between air pollution and physical activity, which finds that an increase in air pollution discourages respondents’ time spent on MVPA [14], daily step counts [26], and outdoor physical activities [35]. In our study, we found that the 10-unit increase in AQI was linked with decreasing by 5.94 daily minutes of MVPA, and a 10 µg/m³ increase in PM_2.5_ and PM_10_ air pollution was linked with a reduction of 7.51 and 13.18 MVPA daily minutes of MVPA, respectively. This finding is consistent with previous research reporting that air pollution was found to be negatively associated with time spent on moderate to vigorous physical activity (MVPA), in which one unit (44.72–56.6 µg/m³) PM_2.5_ increased in air pollution will discourage 32.45 weekly minutes of MVPA among Chinese college students [14, 17]. Similarly, our recent study over 340 Chinese university students using an objective accelerometer linked one level AQI and 10 µg/m³ hourly average PM_2.5_ increase in air pollution with a decline in total hourly minutes of MVPA of 0.083 and 0.021, a decline in walking steps in hourly steps of 8.8 and 2.2, respectively [26]. In addition, other air pollution indicators (i.e., O_3_, NO_2,_ and SO_2_) were examined for the associations between air pollution and physical activity [20, 22]. The results of the other air pollution indicators were found to be negatively associated with older adults’ time spent on daily walking step count [16] and adults’ lower overall physical activity [22]. Inconsistent with this study, one study reported that PM_2.5_ level and the ambient temperature had no impact on physical activity in Beijing of 40 Han Chinese adults [27]. Another study showed that Chinese adults (the user of an exercise app) ran and walked the same distance and duration under different air conditions [21]. A possible explanation for this difference could be that the difference between the participants and people may not change their physical activity routines in response to air pollution until the air quality is up to a high level [36]. For example, in our previous study, university students in China did not discourage their physical activity when air pollution was at PM_2.5_ in 68.78 µg/m^3^; however, their physical activity decreased rapidly when PM_2.5_ was up to 165.13 µg/m^3^ [14].

Our results suggest that AQI, PM_2.5,_ and PM_10_ increases at one level were associated with children’s higher sedentary behavior in one day. This study is consistent with previous studies showing the positive correlation between air pollution and sedentary behavior [37–39]. This finding suggests that a daily AQI, PM_2.5,_ and PM_10_ increase was linked with increased sedentary behavior by 15.76, 21.12, and 19.87 min in one day, respectively. Consistent with our previous researches, an increase in air pollution concentration in PM_2.5_ by 81.16 µg/m³ which was associated with an increase in total weekly hours of SB by 6.24 h among a large sample (12,174) of university freshmen in China based on a cohort study survey [39] and an increase in air pollution concentration in PM_2.5_ by 56.6 µg/m³ which was associated with an increase in total daily hours of SB by 0.71 h among older adults (university retirees) in China based on a follow-up survey [13]. A previous study reports that on days with better AQI levels, people spent approximately 20 min lesser sedentary time than on moderately and heavily polluted days. PM_2.5_ pollution was associated with an approximately 45 min increase in SB, but the concentration of PM_10_ and O_3_ were not associated with SB based on 3,270 Chinese users’ wrist-worn activity trackers data [37]. However, contrary to the above result, a previous publication reported that moderate-to-severe air pollution (AQI > 150) was not associated with daily television use among residents in Shanghai, China [40]. A possible reason for this difference could be that the residents’ SB behavior is different from our children’s samples. Moreover, a recent systematic scoping review examined the associations between sedentary behavior and air pollution [36]. The results of the review showed that the pattern was not consistent. Overall, findings reported increased sedentary-related behavior in relation to air pollution. One possible explanation for this pattern is that people are often advised to refrain from going outside on highly polluted days [41]. Because of inconsistent results in this emerging area, more investigations are necessary to fully estimate the impact of air pollution on sedentary-related behavior among different groups.

There are several potential pathways in which air pollution may discourage physical activity and increase sedentary behavior. First, media alerts about air pollution may change people’s decisions about outdoor physical activity. A previous study indicated that daily changes in air pollution levels and media alerts informing the public about risky air pollution levels might decrease outdoor activity among adults with asthma [24]. Moreover, a recent study reported that an increase in media alerts was associated with lower walking time in South Korea [36]. Similarly, the impact of air pollution alerts on cyclist behaviors was reported in Sydney [42], Australia, and found a 14–35% decrease in bicycle use. Ward and Beatty investigated the relationship between air pollution alerts and physical activity [43]. During alert days, participants, especially those whose ages are above 65 years old, would decrease vigorous physical activity by 82%. In addition, several previous studies [40, 44, 45] reported that air pollution might discourage people from engaging in physical activity due to media alerts. Second, the smog appearance could discourage people from doing more physical activity. For example, hazy weather perception was associated with a reduction in cycling and walking traveling by 68% and 64% among Chinese adults [46]. A likely explanation is that people are concerned about the negative associations of the increase in inhaling air pollution due to physical activity. For instance, a previous study reported that the inhalation rates of air pollutants increased with an increase in the level of activity among participants [47]. Similarly, commuters engaging in walking or cycling had higher inhaled doses of air pollutants than those using motorized transport [48]. Third, children and older adults are more susceptible to air pollution and may respond more to air pollution. In this study, all participants were recruited from children. Early childhood is a specific period of habits formed, and family lifestyle routines are open to changes and adaptations [7]. Parents and teachers may teach their children to do more physical activities in the presence of air pollution which will bring additional risks. As a result, children engage in less physical activity and spend more time on sedentary behavior during air pollution days.

This study has several strengths. First, to our best knowledge, it is the first study to use objective data to investigate the association between air pollution and physical activity and sedentary behavior. Second, we used the accelerometer to measure physical activity and sedentary behavior among children. The observed data of air pollution, physical activity, and sedentary behavior presented significant and precise relationships between each other. Most existing previous studies [13–15, 17–21, 24, 25] on the association between air pollution and physical activity or sedentary behavior have used subjective methods, allowing for uncontrolled confounding bias due to self-report. Third, because air quality levels varied daily, we objectively measured daily data would be able to analyze within-child associations between daily air quality and daily physical activity and sedentary behavior. Fourth, this study lies in its longitudinal study design. We performed the individual fixed-effect models to eliminate confounding bias from factors that remained constantly within-participant over time. Nevertheless, there are a few major limitations to this study that should be noted. First, we were not able to distinguish outdoor and indoor, home and kindergarten physical activities. Second, we assessed the concentration of air pollution data from the monitoring station nearest to their kindergarten. We didn’t assess daily AQI through the individual’s residential address and we didn’t use the hourly full-coverage PM_2.5_ dataset [49]. Third, air pollution exposures may play a significant role in terms of lagged or cumulative effects, which may be linked to the toxic effects manifested following extremely-polluted days. Our current study was limited as the lack of estimation of the lag/cumulative effects, which should be included in our future studies. Fourth, we did not collect parents’ socioecnomic status, air pollution exposure and the duration of physical activity may be associated with day of week and parents’ socioeconomic status, therefore, this is a significant confounder, and future studies should control the confounder and figure out a clearer relationship between air pollution and health behavior among children. Fifth, all participants were recruited from a convenience sample. Children from one kindergarten cannot represent all children in China, therefore, limiting the generalizability of the study’s findings. Future studies are warranted to produce more generalized estimates.

## Conclusion

This study examined the longitudinal relationship between daily air pollution variation and daily behavioral modification by using objectively-assessed PA and SB among children in China. Ambient air pollution concentration of AQI, PM_2.5,_ and PM_10_ was negatively associated with daily physical activity but was positively linked with daily sedentary behavior. Children are more susceptible to air pollution exposure.

## Data Availability

The datasets generated and/or analyzed during the current study are not publicly available due to confidentially reasons, but are available from the corresponding author on reasonable request.
